# Case report: 2 cases of cardiac arrest caused by rhino-cardiac reflex while disinfecting nasal cavity before endonasal transsphenoidal endoscopic pituitary surgery

**DOI:** 10.1186/s12871-021-01240-w

**Published:** 2021-01-13

**Authors:** Wen Wang, Hongwei Cai, Huiping Ding, Xiaoping Xu

**Affiliations:** Department of Anaesthesiology, Xiangya Hospital Central South University, Changsha, 410008 Hunan China

**Keywords:** Trigeminal-cardiac reflex, Rhino-cardiac reflex, Cardiac arrest, Disinfect;endoscopic transsphenoidal removal of pituitary adenomas

## Abstract

**Background:**

Trigeminal-cardiac reflex (TCR) is a brainstem vagus reflex that occurs when any center or peripheral branch of the trigeminal nerve was stimulated or operated on. The typical clinical manifestation is sudden bradycardia with or without blood pressure decline. The rhino-cardiac reflex which is one type of TCR is rare in clinical practice. As the rhino-cardiac reflex caused by disinfection of the nasal cavity is very rare, we report these two cases to remind other anesthesiologists to be vigilant to this situation.

**Case presentation:**

This case report describes two cases of cardiac arrest caused by rhino-cardiac reflex while disinfecting nasal cavity before endoscopic transsphenoidal removal of pituitary adenomas. Their heart rate all dropped suddenly at the very moment of nasal stimulation and recovered quickly after stimulation was stopped and the administration of drugs or cardiac support.

**Conclusion:**

Although the occurrence of rhino-cardiac reflex is rare, we should pay attention to it in clinical anesthesia. It is necessary to know the risk factors for preventing it. Once it occurs, we should take active and effective rescue measures to avoid serious complications.

## Background

Trigeminal-cardiac reflex (TCR) is a brainstem vagus reflex that occurs when any center or peripheral branch of the trigeminal nerve was stimulated or operated on. The typical clinical manifestation is sudden bradycardia with or without blood pressure decline. The oculocardiac reflex and the gallbladder-heart reflex are more common. Enough attention has been paid to them during the perioperative period. However, the related literature of rhino-cardiac reflex is rare. Here are two cases of cardiac arrest caused by rhino-cardiac reflex while disinfecting nasal cavity before endoscopic transsphenoidal removal of pituitary adenomas. As the rhino-cardiac reflex caused by disinfection of the nasal cavity is very rare, we share these two cases to remind other anesthesiologists to be vigilant in this situation.

### Case one

A 66-year-old man,70 kg,175 cm, BMI22.9 kg/m^2^, was admitted for saddle area tumor on June 23rd, 2020. His chief complaint was blurred vision for more than 2 months and aggravating for more than 10 days. He had been suffering from hyperthyroidism for more than 10 years and was treated with iodine-131 and taking thyroxine irregularly. Preoperative blood routine test, biochemical examination, ECG were all normal. Thyroid function showed abnormal, such as free thyroid hormone was 8.28 pmol/L (reference range:12-22 pmol/L), high-sensitivity thyrotropin was 57.57mIU/L (reference rang:0.27–4.2mIU/L). He had a long history of smoking but no lung or heart disease. He did not use anesthesia auxiliary drugs before the operation. On the day of operation, the patient was routinely monitored for ECG, BP, and SpO2. His blood pressure was 125 / 70 mmHg, heart rate (HR) was 80 beats/min with a regular rhythm, and SpO_2_ was 95%. Midazolam 3 mg, cisatracurium 14 mg, etomidate 20 mg, sufentanil 35μg was given intravenously. Tracheal intubation was performed and mechanical ventilation was started. At this time, the patient’s BP was 99/58 mmHg, HR was 63 times/min, SpO2 was 100%. Sevoflurane and propofol were administered to maintain anesthesia. After the patient’s position was placed, iodophor disinfectant was dripped in his bilateral nasal cavity (34 min away from anesthesia induction). At this very moment, HR suddenly slowed down and dropped to 0 times/min, and the arterial blood pressure was 63/28 mmHg, SpO2 was 100%. When HR slowed down, atropine 0.5 mg was given intravenously and the surgeon was immediately informed to stop the operation. CPR was performed immediately at the very moment of cardiac arrest. After 30 s, the heart rate rose slowly to 63 beats/min, the blood pressure was 120/56 mmHg, and SpO2 was100%. After the patient’s vital signs were stable. The operation was canceled for the request of the patient’s family. The patient regained consciousness 30 min later in the PACU. His heart rate was maintained between 62 and 68. After 1 day of observation in the ICU, he was transferred back to the ward. The patient was clear in consciousness and his language, memory, orientation, and intelligence were all normal. His blood routine and biochemical tests had no obvious abnormality. The patient was discharged 3 days later.

### Case two

A 37-year-old man,65 kg,176 cm, BMI21.0 kg/m^2^, was admitted for saddle area tumor on July 14th,2020. His chief complaint was a vision loss for 5 years and a headache for 3 days. His previous history was negative. Preoperative blood routine test, biochemical examination, ECG were normal. After entering the operating room, his vital signs were monitored. Blood pressure was 105 / 55 mmHg, HR was 80 beats/ min and SpO2 was 100%. Midazolam 3.5 mg, cisatracurium 12 mg, etomidate 20 mg, and sufentanil 35μg were administered intravenously. The anesthesia induction and tracheal intubation were successful. His blood pressure was 95/49 mmHg, HR was 85 beats/ min and SpO2 was 100%. Sevoflurane and propofol were given to maintain anesthesia. After the patient’s position was placed, iodophor disinfectant was dripped in his bilateral nasal cavity. (30 min away from anesthesia induction). The patient’s heart rate dropped suddenly. The monitor showed a value of 31 beats/min. The ECG showed that the heart rate was irregular. We found a pause of more than 3 s. His BP was 97/52 mmHg and SpO2 was 100%. After 0.5 mg atropine was given intravenously for 30 s, his HR gradually returned to 60 beats/min, blood pressure was 99/59 mmHg and SpO2 was 100%. After the vital signs were stable, his operation was continued. There was no significant change during the procedure. Vital signs were stable. His heart rate was maintained at 60–90 beats per minute. He regained consciousness rapidly in PACU. The patient was discharged on the 8th day after the operation.

## Discussion and conclusion

Common causes of HR slowdown during operation under general anesthesia include circulation suppression by anesthetic agents, inadequate volume, vagus nerve stimulation during intubation, and parasympathetic nervous system reflex induced by traction stimulation during surgery. The slow HR caused by anesthetics and volume insufficiency generally occur slowly and accompanied by a sharp drop in blood pressure [1]. The two cases are not consistent with the above situation. Both cases occurred about 30 min after induction, and the hemodynamics was stable. The effects of tracheal intubation, anesthetic, and vasoactive agents on the heart rate could be excluded. There was no abnormality in two patients’ ECGs before the operation. Their heart rate all dropped suddenly at the very moment of nasal stimulation and recovered quickly after stimulation was stopped and the administration of drugs or cardiac support. Therefore, the trigeminal nerve reflex was considered the cause of cardiac arrest.

The trigeminal nerve reflex (TCR) is a brainstem vagal reflex that occurs during stimulation or manipulation of the trigeminal nerve center or any of its peripheral branches. The typical clinical presentation is sudden bradycardia with or without hypotension, respiratory arrest, or gastrointestinal peristalsis [2]. TCR is usually defined as a drop in HR and MABP above 20% of the baseline [3]. Rhino-cardiac reflex is a type of TCR that can be triggered by touching any branch of the trigeminal nerve. The distribution of mechanical stimulation receptors in nasal mucosa is uneven in the whole nasal cavity, and the most sensitive sites to mechanical pressure are mainly located in the posterior nasal mucosa [4]. TCR most commonly occurs in maxillofacial surgery and direct traction stimulation of the trigeminal nerve branches during neurosurgery. TCR has been reported occasionally in skull base surgery and maxillofacial surgery [5, 6]. It commonly happens in endoscopic sinus surgery with nasal packing or transsphenoidal approach for resection of pituitary adenomas [7]. But only one case has been reported because of nasal disinfection [1]. In this case, the patient’s heart rate dropped to 23 beats per minute when the surgeon performed nasal disinfection when 15 min after induction of anesthesia, and gradually returned to normal after administration of 0.5 mg atropine.

Previous studies have shown that the risk factors of TCR include children, hypoxemia, hypercapnia, water-electrolyte disorders, calcium channel blocker, opioids (especially fentanyl), preoperative use of β-receptor blockers [8]. In our cases, both patients were in mechanical ventilation, which could exclude the influence of hypoxemia and hypercapnia. Besides, patients undergoing transsphenoidal resection of pituitary tumors were given opioids during anesthesia induction. These two patients were not exceptional. They also had sufficient depth of anesthesia. No β-receptor blockers and calcium channel blockers were used. So there was no apparent cause of cardiac arrest in either case.

Although previous studies have shown that stopping surgery during TCR generally restores heart rate. If conventional methods fail to do so, patients with sudden cardiac arrest need cardiac life support. It’s a huge challenge for anesthesiologists and surgeons. Disha Awasthi et al. reported a case of death due to cardiac arrest of TCR during nasal packing operation [9]. Disha Awasthi et al. reported a case of death due to cardiac arrest of TCR during nasal packing operation in which vasoactive drugs were not used [9]. So it is very important to prevent rhino-cardiac reflex. First, we need to pay enough attention to the rhino-cardiac reflex. The vital signs of the patient should be closely monitored not only during the operation but also during the preoperative nasal disinfection preparation. Also, we should be more vigilant to patients with primary heart disease, such as sinus bradycardia, atrioventricular block, coronary heart disease, and so on. Drugs such as dexmedetomidine, calcium channel blocker, and β-receptor blockers, etc. that slow down heart rate should be avoided. Topical anesthesia for nasal mucosa before nasal surgery may be an effective preventive measure. Studies have shown that local infiltration anesthesia or nerve block is considered to prevent and treat TCR [10]. However, lidocaine combined with epinephrine did not prevent TCR in some studies [11]. Preoperative administration of anticholinergic may be another way to prevent TCR, but no literature has confirmed that preoperative intramuscular administration of atropine or glycopyrrolate is an effective measure to prevent TCR in adult patients [8]. In a systematic review of the effects of anesthesia on TCR, Cyrill Meuwly et al. found that the risk of TCR under shallow anesthesia was 1.2 times higher than deep anesthesia [12]. And Robert W. Arnold et al. showed that deep anesthesia has a protective effect on the oculocardiac reflex and intraoperative bispectral index (BIS) monitoring can reflect the depth of anesthesia [13]. Therefore, it is necessary to apply BIS monitoring. In anesthesia management, we should maintain an appropriate depth of anesthesia, ensure ventilation oxygenation, avoid anesthesia shallow, hypoxemia, and hypercapnia, maintain water-electrolyte balance, and avoid low blood volume and electrolyte disorder. These are effective measures to prevent rhino-cardiac reflex. When TCR happens, we need to stop nasal operation immediately, give anticholinergic drugs when necessary to raise heart rate, and perform cardiopulmonary resuscitation rapidly when cardiac arrest occurs.

In conclusion, although the occurrence of rhino-cardiac reflex is rare, we should pay attention to it in clinical anesthesia. It is necessary to know the risk factors for preventing rhino-cardiac reflex. Once the rhino-cardiac reflex occurs, we should take active and effective rescue measures to avoid serious complications.

## Data Availability

Data and material are available from the corresponding author on reasonable request.

## Abbreviations

TCR: Trigeminal nerve reflex
BP: Blood pressure
SPO2: Pulse oxygen saturation
PACU: Post anesthesia care unit
BIS: Bispectral index
